# Reduced Computed Tomography for Appendicitis in Children after Implementation of Next-day Surgery Clinic Follow-up

**DOI:** 10.1097/pq9.0000000000000641

**Published:** 2023-03-13

**Authors:** Sydney Ryan, Nanette C. Dudley, Jeff E. Schunk, Cindy Weng, David E. Skarda, Eric W. Glissmeyer

**Affiliations:** From the * Division of Pediatric Emergency Medicine, Department of Pediatrics, University of Utah, Salt Lake City, Utah; † Department of Pediatrics, University of Utah, Salt Lake City, Utah; ‡ Division of Pediatric Surgery, Department of Surgery, University of Utah, Salt Lake City, Utah.

## Abstract

The use of computed tomography (CT) in the emergency department (ED) evaluation of pediatric patients for suspected appendicitis can be safely reduced. However, published examples of reduced CT use also report increased MRI utilization, ED length of stay, hospitalization rates, and in-ED surgical consultation. In addition, previous studies recommended follow-up for undifferentiated abdominal pain, yet none with pediatric surgeons. Therefore, we implemented a diagnostic algorithm that includes an option for next-day surgery clinic follow-up in cases where uncertainty remains after appendix ultrasound (US) to reduce CT utilization without increasing hospital-based resources.

**Methods:**

We implemented a diagnostic algorithm in January 2014. We retrospectively identified 4,577 patients who underwent an evaluation for suspected appendicitis from January 2012 to September 2015. CT utilization was compared before and after implementation using Statistical Process Control. In addition, we evaluated secondary outcomes, including US utilization, hospital admission, surgery clinic follow-up, ED surgery consultation, ED return visits within 7 days, and ED length of stay.

**Results:**

Following the implementation of the algorithm, CT utilization decreased significantly from 13.8% to 6%. Forty-eight patients were evaluated the next day in the optional pediatric surgery clinic for 21 months after implementation. There was no significant change in US utilization, hospital admission, ED surgery consultation, ED return visits within 7 days, or ED length of stay.

**Conclusion:**

We achieved decreased CT utilization without an increase in the utilization of other hospital-based resources after implementing a pediatric appendicitis evaluation algorithm that includes the option for next-day pediatric surgery clinic follow-up.

## INTRODUCTION

### Problem Description

Appendicitis is the most common surgical emergency in children. Pediatric patients with abdominal pain frequently present to the emergency department (ED) for evaluation of suspected appendicitis.^1,2^ The diagnostic assessment of suspected appendicitis often employs a combination of laboratory, computed tomography (CT), and ultrasound (US)^3^ evaluation. CT scan use for children with abdominal pain was 15% nationally in 2008^4^; by 2012, CT use was between 16% and 55% in children with abdominal pain.^5,6^ Ionizing radiation exposure concerns and the widespread use of diagnostic ultrasonography led to a recent decline in CT use in children diagnosed with appendicitis.^7^ Others have described using a limited right lower quadrant MRI to reduce ionizing radiation exposure.^8,9^

### Available Knowledge and Rationale

Variability in diagnostic approach motivated several studies implementing pediatric appendicitis scoring systems to risk-stratify patients. These studies emphasize the selective use of US, resulting in reduced CT utilization.^2,10–13^ Recent studies have successfully reduced CT rates in children evaluated for suspected appendicitis with diagnostic protocols within single institutions^10,11^ and in regional care systems of community hospitals.^12^ However, these gains in reduction of CT use resulted in increased utilization of MRI, longer ED length of stay (over 5 hours),^10^ increased in-ED surgical consultation (70%),^11^ and high rates of admission (above 50%).^2^ These authors and others^13^ have recommended follow-up for undifferentiated abdominal pain, yet none with pediatric surgeons. We aimed to decrease CT utilization without increasing the use of hospital-based resources by implementing a diagnostic algorithm in our ED for patients evaluated for suspected appendicitis that included a novel approach of offering a pediatric surgery clinic option for next-day evaluation in cases of diagnostic uncertainty. We created this next-day follow-up clinic option as an alternative to further imaging workup in patients with diagnostic uncertainty.

### Specific Aims

Our primary outcome measure and objective was to reduce abdominal CT scans by 50% in ED patients with suspected appendicitis using a diagnostic algorithm incorporating the newly developed weekday, next-day pediatric surgery clinic follow-up for select cases. We aimed to achieve this reduction within 3 months of algorithm implementation and sustain this improvement for at least 1 year. Balancing measures used to determine whether a change in CT utilization caused unwanted changes in other parts of our system included US utilization, hospital admission, surgery clinic follow-up utilization, in-ED surgery consultation, ED return visits within 7 days, and ED length of stay. We hypothesized that after introducing the diagnostic algorithm and utilizing the novel next-day surgery clinic follow-up, CT scan use would decrease without significant changes in balancing measures.

## METHODS

### Context

This report describes a quality improvement quasi-experimental study at a single academic pediatric ED. The institution review board at the University of Utah approved the study. Revised Standards for Quality Improvement Reporting Excellence (SQUIRE 2.0) guided study design and manuscript writing.^14^ We collected all data from the enterprise data warehouse (EDW) of Intermountain Healthcare. All encounters occurred at Primary Children’s Hospital, a regional referral center, and a level-one trauma center with an annual ED census of 44,000 and an admission rate of 23%. The negative appendectomy rate varies between 1% and 4% at this site, consistent with the national rate.^15^ Before this study, we implemented a US program available 24 hours. After implementing a US protocol with standardized reporting language, the imaging department decreased equivocal US interpretations.^16^ Based on US findings, this change resulted in an increase in pediatric patients undergoing an appendectomy. Therefore, our hospital does not use MRI for ED evaluation of suspected appendicitis.

### Description of the Intervention

**Fig. 1.**
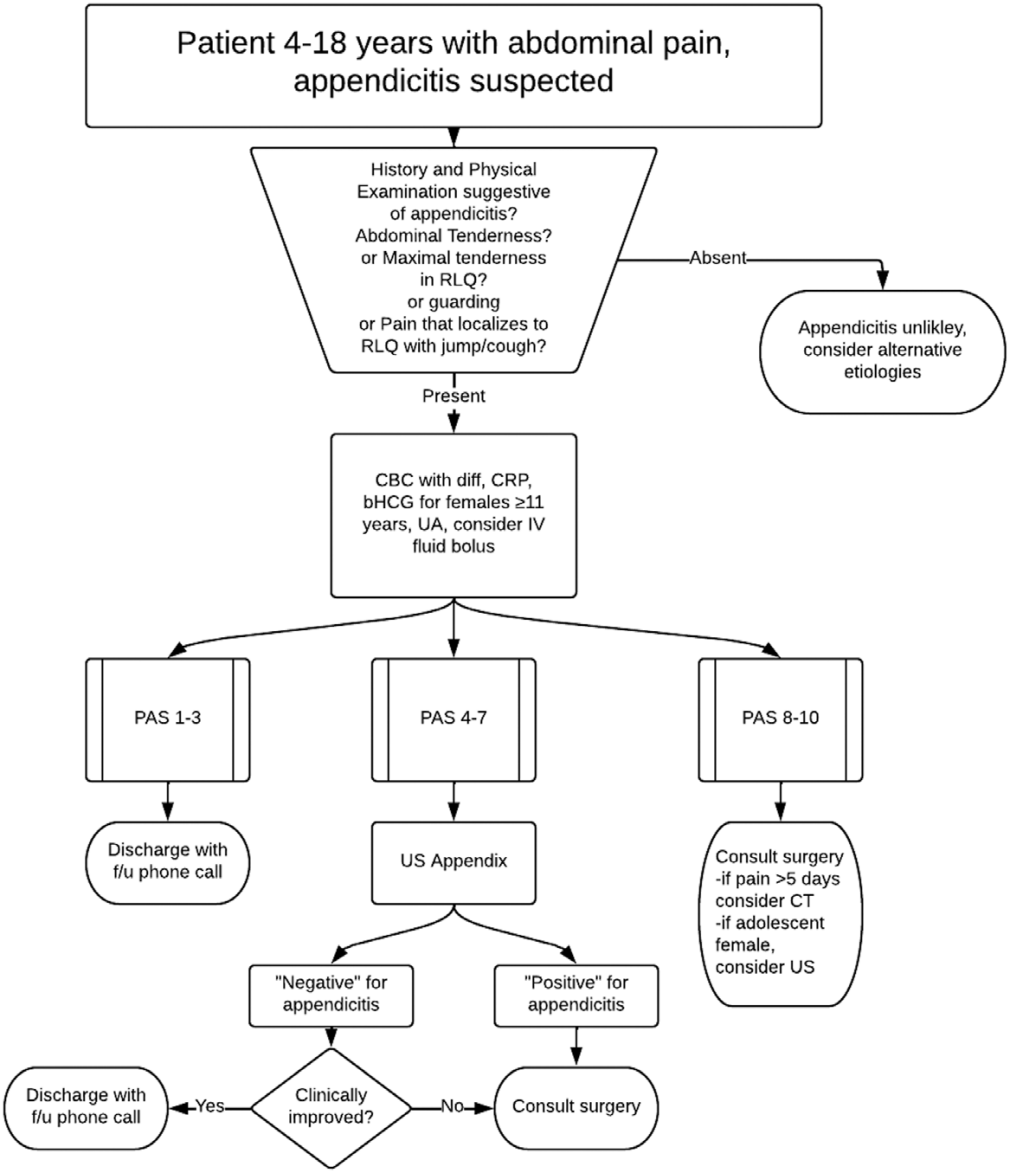
Pediatric appendicitis diagnostic algorithm used in this study.

Members of pediatric emergency medicine, surgery, and radiology divisions formed a team in January 2013. They developed a diagnostic algorithm for suspected appendicitis in October 2013 that begins using history, physical examination, and laboratory evaluation with a US-first approach for imaging^17^ (Fig. 1). Providers use the Pediatric Appendicitis Score (PAS) for disposition and imaging decisions.^18^ For a PAS of 8–10, the algorithm recommends surgical consultation before additional imaging, allowing for shared decision-making between surgical and emergency medicine providers and for operative management of appendicitis without any imaging tests performed. We introduced the diagnostic algorithm and provider education in January 2014. This education included introducing and discussing the algorithm during meetings of the Divisions of Emergency Medicine, Surgery, and Radiology. We placed a copy of the algorithm at each ED physician workstation. In cases of diagnostic uncertainty with equivocal clinical, laboratory, and/or appendix US evaluation and persistent right-lower quadrant tenderness, the algorithm offers an alternative option to CT imaging: next-day surgery clinic follow-up on weekdays. Consulting surgery nurse practitioners gave instructions to patients referred to this clinic and asked families to call in the morning for persistent symptoms. Those with persistent pain were seen as add-on visits in the general surgery clinic. Due to our focus on outcomes at our institution, we did not include patients transferred from outside hospitals with CT results. We did not directly measure adherence to the algorithm. After implementation, we reeducated ED providers in June 2014 on additions to the algorithm recommending consideration of CT if pain had persisted for more than 5 days in a patient with a PAS score of 8–10 and consideration of US in adolescent females with PAS 8–10 before surgical consultation.

### Study of the Intervention and Measures

We retrospectively identified patients undergoing an evaluation for suspected appendicitis using a surrogate definition—the performance characteristics we described previously.^17^ The surrogate definition combines chief complaint, laboratory, and text data from clinical notes and the acquisition of a right lower quadrant US.

We collected data from January 1, 2012, to September 30, 2015, using the EDW of Intermountain Healthcare. In January 2014, we implemented a diagnostic algorithm through presentations of the new algorithm and follow-up clinic availability. In addition, we posted physical copies of the algorithm in the ED. We compared data from the 24-month preimplementation period (January 2012–December 2013) to 21-month postimplementation period (January 2014–September 2015). Other than the reeducation efforts described above, we made no other interventions regarding CT and US imaging practices, surgical service consultation availability, and inpatient observation availability. During the study, a stable group of pediatric surgery nurse practitioners was the first call for ED surgical consults.

Data collected included demographics, results of abdominal or abdomen/pelvis CT scans, right-lower quadrant US, disposition status (admitted versus discharged to home), the occurrence of a pediatric surgery clinic visit within 48 hours of the ED encounter, the presence of a surgical consultation note in connection with the ED encounter, return ED encounter within 7 days of the index ED encounter, and total ED length of stay. In addition, we analyzed balancing measures for any change in additional resource utilization, including the number of surgery consults in the ED, ED return visits within 7 days, and median length of stay in the ED.

### Analyses

We applied our surrogate definition to our EDW to identify the patients evaluated for suspected appendicitis before and after implementing the diagnostic algorithm. We analyzed CT utilization rate using a Statistical Process Control p-chart with upper and lower control limits at ±3 standard deviations and average utilization rates preimplementation and postimplementation of the appendicitis diagnostic algorithm. We compared balancing measures and a subanalysis of our primary outcome preimplementation to postimplementation using chi-square analyses in SAS 9.4.

### Ethical Considerations

We enrolled subjects retrospectively in this study conducted as a quality-improvement effort. In addition, we protected patient data on a secure server and deidentified patient data. Therefore, the Intermountain Healthcare and University of Utah institutional review boards (IRB 79788) considered this study exempt from full review.

## RESULTS

### Characteristics of Study Subjects Evaluated for Suspected Appendicitis

**Fig. 2.**
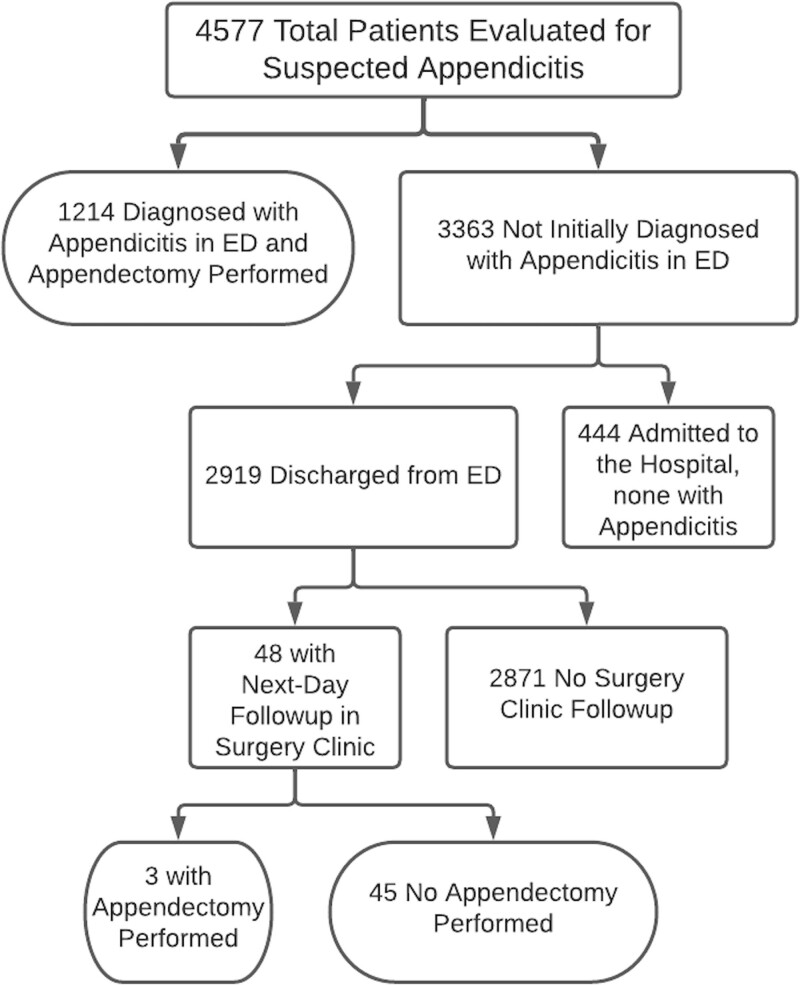
CONSORT diagram of patients evaluated for suspected appendicitis and their outcomes.

We identified 4,577 children evaluated for suspected appendicitis from January 2012 to September 2015; their outcomes are shown in Figure 2. There were 2,177 children in the preimplementation period, and 2,400 children in the postimplementation period. Appendicitis rates were similar preimplementation to postimplementation at 28% versus 26%, respectively, and the median age (10) and biological gender (54% female) did not change during the study (Table 1).

**Table 1.** Demographic Data of 4,577 Patients Evaluated for Suspected Appendicitis Preimplementation versus Postimplementation

	Preimplementation, n = 2,177	Postimplementation, n = 2,400	*P*
Appendicitis, n (%)	600 (28)	614 (26)	0.36
No appendicitis, n (%)	1,577 (72)	1,786 (74)	0.57
Age, y, median (q1–q3)	10 y (7–13)	10 y (7–13)	0.12
Female, n (%)	1,197 (55)	1,294 (54)	0.46

### Primary Outcome: CT Utilization

**Fig. 3.**
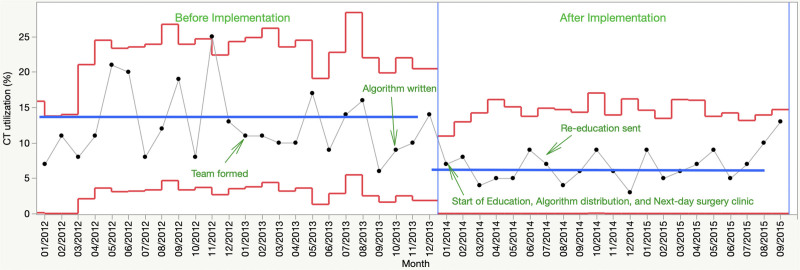
SPC P-chart of CT utilization decrease from 13.8% preimplementation to 6% postimplementation. The blue horizontal line is the monthly mean CT utilization rate. The red lines are ±3 standard deviations above and below the monthly mean. Events in algorithm development and implementation appear on the chart at the dates they occurred. SPC indicates statistical process control.

After implementation of the diagnostic algorithm, CT utilization decreased as defined by having all points on the same side of the preimplementation average CT utilization (Shewhart Rule 2)^19^ from 13.8% to 6% (Fig. 3), and US use did not change (61.5% versus 62.5%). We annotated key steps in the algorithm development and implementation in Figure 3. Baseline data before implementation did not demonstrate any special cause variation. However, the mean CT utilization rate remained below the preimplementation mean for 21 months following implementation, indicating special cause variation after introducing the diagnostic algorithm.

Of the 4,577 patients evaluated for suspected appendicitis, 1,214 (26.5%) were diagnosed with appendicitis in the ED: 600 preimplementation and 614 postimplementation. Of those diagnosed with appendicitis, CT utilization also decreased [62 (10.3%) pre to 34 (5.5%) post, *P* = 0.0042], and US use remained unchanged [409 (68%) pre to 396 (64%) post, *P* = 0.82]. Of 3363 (73.5%) patients evaluated for suspected appendicitis and not diagnosed with appendicitis in the ED, CT utilization also decreased [238 (15%) pre to 110 (6%) post, *P* < 0.0001] and hospitalization remained unchanged [206 (13%) pre to 238 (13%) post, *P* = 0.97].

### Resource Utilization Results

Of the 3,363 not initially diagnosed with appendicitis in the ED, 2,919 were discharged home, and Table 2 describes measures of their resource utilization. Forty-eight (3.1%) had next-day surgery clinic follow-up after implementation, compared with 12 (0.9%) preimplementation (*P* < 0.0001). Of those 48 patients seen in the next-day surgery clinic, 3 underwent appendectomy after that visit because of persistent RLQ tenderness. These 3 patients were pathologically negative for appendicitis. None of the 48 patients had CT at the next-day surgery clinic. There were no significant changes in the number of surgery consults in the ED, ED return visits within 7 days, or median length of stay in the ED. None of the 109 ED return visits after implementation had appendicitis (Table 2).

**Table 2.** Resource Utilization among Patients Discharged from the ED after Evaluation for Suspected Appendicitis (N = 2,919)

	Preimplementation (%), n = 1,357	Postimplementation (%), n = 1,562	*P*
Surgery clinic follow-up	12 (0.9)	48 (3.1)	<0.0001
Surgery consultation in ED	125 (9.2)	114 (7.3)	0.06
ED return visit within 7 d	86 (6.3)	109 (7.0)	0.49
Median ED length of stay, hours (SD)	4.6 (1.9)	4.3 (1.7)	0.10

## DISCUSSION

Within our institution, using a diagnostic algorithm to evaluate patients with suspected appendicitis with a novel next-day pediatric surgery clinic follow-up option decreased abdominal CT utilization without changes in US utilization for 21 months after algorithm implementation. This finding met our primary objective and aim. In addition, we reduced CT utilization without changing other measures of resource utilization, including admission rate, ED length of stay, and in-ED surgery consults. There was an increase in next-day surgery clinic visits of less than 1 visit per week.

This work reinforces the benefit of a care pathway in diagnosing appendicitis and describes a novel collaborative approach of next-day surgery clinic follow-up in select cases to manage diagnostic uncertainty. When diagnostic uncertainty persists for surgical causes of abdominal pain and patients do not need admission to the hospital for observation, next-day clinic follow-up with a pediatric surgeon is a significant benefit to pediatric patients and their families. Pediatric surgeons can examine the patient, obtain repeat laboratory or imaging tests, and decide whether to perform appendectomy, all outside the emergency setting with its associated high cost of care. This study includes all patients evaluated for suspected appendicitis, including those diagnosed with appendicitis and those ultimately found to have other diagnoses. Few other studies^2,14^ include all patients evaluated for suspected appendicitis. This study is the first to use an administrative data-based surrogate definition to identify all patients evaluated for suspected appendicitis in place of prospective data collection. In addition, this work included more than 10 times the number of patients in those similar studies.

In cases that can be diagnostically challenging, such as incomplete visualization of the appendix on US or equivocal laboratory findings, a next-day surgery clinic follow-up option is an alternative to further imaging and a benefit to patients and ED providers. We reviewed return ED visits within 7 days. We found no statistically significant increase in return visits, which supports the use of the follow-up surgery clinic option as an alternative to CT and other advanced imaging modalities. Others have proposed MRI as a radiation-sparing alternative to CT in cases of post-US diagnostic uncertainty. However, this comes at an increased cost, with a 22% longer ED stay than reported in this study.^10^ Previous studies with similar appendicitis rates and CT utilization to this study reported higher hospitalization rates, 51%^2^ compared with 36% in this study, highlighting the admission reduction achieved when offering a next-day surgery clinic follow-up option. Hospital admission is sometimes necessary but costly and inconvenient solution to diagnostic uncertainty.

This study strikes a novel balance by offering pediatric surgery clinic follow-up in cases of diagnostic uncertainty, reducing CT use without increasing the use of other hospital resources. Next-day surgery clinic follow-up offers emergency physicians an additional option to manage diagnostic uncertainty in patients with persistent abdominal pain not needing hospitalization. Next-day surgery clinic provides the prescreened child with persistent abdominal pain access to pediatric surgeons outside the emergency setting and avoids the high costs of ED care. The PAS has become a prominent part of the presentation or consultation during provider communication. The initial surgical consultation and examination occur with the surgical Advance Practice Provider team and the surgeon. This Advance Practice Provider team consists of nurse practitioners employed at our site and do not rotate, allowing for familiarity with the discussion around PAS and awareness of the next-day clinic follow-up option. Medical and surgical teams reference the diagnostic algorithm during shared decision-making and with patients and families. We reserve appendix CT for prolonged pain ≥5 days and when requested by the surgery team. As measured by ED consultation by surgery without admission to the surgery service and diagnosis of appendicitis, surgical consultation was 7% in our study. AlFraih et al’s^11^ work demonstrated similar low utilization of CT, a higher admission rate, and a much higher surgical consultation rate of 70%. Even if we include the 26% of patients in our study diagnosed with appendicitis (Table 1), our surgical consultation rate was 33%, or less than one-half of that reported previously. Many patients may present with evolving or early appendicitis symptoms and have low PAS scores. These patients may be good candidates for a follow-up surgery clinic. The option of a next-day surgery clinic can help reduce CT utilization and radiation exposure for children who are not diagnostically clear.

Limitations of this study included those inherent to retrospective research using administrative data, including an inability to capture the PAS assigned to a subject reliably. Our electronic health record did not codify these data during the study period. Given the 79.8% sensitivity of the surrogate definition, it was possible to have missed a small number of patients suspected of appendicitis who presented with atypical chief complaints and did not undergo appendix US. But as described in that work, referring facilities had previously evaluated with imaging or laboratory workup 12 of the 19 patients missed by the surrogate definition.^17^ The consistency of our surgery nurse practitioners providing initial ED surgical consultation facilitated our algorithm’s adherence but may limit our findings’ generalizability. Emergency medicine physicians and pediatric surgeons do not currently practice in close partnership at all hospitals where children seek care. Yet, next-day pediatric surgery clinic follow-up could be implemented where these specialties exist. We conducted this study using an electronic medical record system that changed in October 2017, and further querying of our EDW will require modifications. We did not gather admission diagnoses, hospital length of stay, or other measures on the 444 children admitted to the hospital after ED evaluation for suspected appendicitis. However, from our validation of the surrogate definition, many subjects without appendicitis had common medical diagnoses for their abdominal pain, such as gastroenteritis, inflammatory bowel disease, or pyelonephritis.^17^ This finding is expected as we designed the surrogate definition to identify patients evaluated for suspected appendicitis beyond those who had imaging tests or appendicitis. We did not directly measure MRI utilization because MRI has never been used for ED patients evaluated for suspected appendicitis at our institution. We did not measure how often we offered the follow-up clinic and, therefore, cannot measure the frequency of patients utilizing it due to symptom resolution. Finally, we did not document provider compliance with the algorithm. Understanding the frequency of follow-up clinic offerings, algorithm compliance, and changes in these over time would help identify opportunities to maximize utilization of the next-day surgery follow-up clinic and reduce imaging further.

### Conclusions

For patients with suspected appendicitis, using a diagnostic algorithm that includes next-day surgery clinic as an option in cases of diagnostic uncertainty decreased the CT scan utilization in patients evaluated for suspected appendicitis by more than 50% from baseline, without increasing the use of other resources, including the performance of appendix US, hospital admission, in-ED surgical consultation, ED return visits, or ED length of stay. These improvements continued for at least 21 months, but further study is necessary to determine the long-term sustainability of these findings, the feasibility of next-day pediatric surgery clinic follow-up in other settings, and the cost savings of our approach.

## DISCLOSURE

The authors have no financial interest to declare in relation to the content of this article.
